# A new secure offloading approach for internet of vehicles in fog-cloud federation

**DOI:** 10.1038/s41598-024-56141-y

**Published:** 2024-03-06

**Authors:** Yashar Salami, Vahid Khajehvand, Esmaeil Zeinali

**Affiliations:** Department of Computer and Information Technology Engineering, Qazvin Branch, Islamic Azad University, Qazvin, Iran

**Keywords:** SO, Security, Fog-cloud, Federation, Computer science, Information technology

## Abstract

The Internet of Vehicles (IoV) plays a crucial role in advancing intelligent transportation systems. However, due to limited processing power, IoV faces challenges in independently handling large volumes of data, necessitating the use of offloading as a solution. Offloading data in wireless environments raises security concerns, highlighting the need for robust data protection mechanisms. This study introduces a secure offloading (SO) scheme within the Fog-Cloud Federation for IoV. The proposed NSO-VFC scheme undergoes both informal and formal analysis using the Avispa tool, demonstrating resilience against active and passive attacks. Performance evaluations indicate that the security measures of NSO-VFC meet acceptable standards compared to similar approaches. Nonetheless, the heightened focus on security incurs higher computational and communication costs than alternative strategies. Simulation experiments using the NS3 tool involve varying numbers of IoVs (50, 70, and 100), revealing that increased IoV density correlates with enhanced packet delivery rates and throughput within the NSO-VFC scheme.

## Introduction

The advancement of distributed systems, such as cloud-fog architecture, enables users to access, share, and modify their data from any location^1–3^. This evolution is further accelerated by the growth of diverse fields within computer science, including the Internet of Things, propelling the rapid development of distributed systems^4^. The Internet of Things is a new technology that has emerged with the progress of the world of science and the needs of today's people for fast communication. Objects in this technology are any device with a sensor to exchange information^5^. These sensors play a crucial role in collecting and transmitting valuable data to the connected devices^6^. One of the key advantages of the Internet of Things is its ability to facilitate interactions with the diverse elements in our environment, including vehicles, individuals, animals, plants, and various other entities^7^. The Internet of Things has given rise to the concept of IoV, where IoV networks function as subsets of mobile networks primarily comprising vehicles^8–10^. IoV networks use wireless communication technology to communicate with their surroundings^11–13^. IoV are fully autonomous; they communicate with each other and create a wireless network^14,15^. Today, vehicles can communicate with the fog layer and send thousands of data to a powerful computing system for processing every moment to transfer data from a series of parameters such as (efficiency, delay, energy, cost, etc.) for Destination selection is used for offloading^16–18^. However, before sending data, it is necessary for the source and destination to acknowledge each other before offloading and to be aware of each other's existence. When the texture and essence of traditional life are transformed into a modern model, social crimes and civil anomalies also take on the color of modernity, and the third person may replace the communication parties. The generality of raw data is endangered. For this reason, it can be said that one of the critical challenges before offloading in fog-cloud environments is authentication.

In this paper, we present a novel approach to elliptic curve cryptography techniques for the IOV in cloud environments. Our primary objective with this proposed method is to deal with active and passive attacks on the issue of offloading. By harnessing the power of elliptic curve cryptography, we aim to enhance the security and privacy of data transmissions within IOV systems operating in cloud environments. This innovative approach offers a robust solution to the challenge of secure offloading, ensuring the confidentiality and integrity of sensitive information exchanged between vehicles and the fog-cloud federation.

### Paper contribution

In this section, we outline the significant contributions of the NSO-VFC scheme, which enhances security in vehicular fog computing environments. The key contributions of the NSO-VFC scheme are as follows.

Resistance to active and passive attacks: The NSO-VFC offers robust security measures to protect the system against both active and passive attacks. By implementing advanced security mechanisms, the scheme ensures the integrity and confidentiality of data transmitted within the vehicular fog computing network.

Mutual authentication in IOV: The NSO-VFC facilitates mutual authentication in the context of the IOV, ensuring that service providers and vehicles can securely authenticate each other before establishing communication. This mutual authentication mechanism adds an extra layer of security to prevent unauthorized access and malicious activities.

Secure session key generation: One of the key features of the NSO-VFC is its ability to generate secure session keys for communication between different entities within the network. By using secure key generation algorithms, the scheme enables secure and encrypted communication channels, protecting sensitive data from unauthorized access or tampering.

Device suggestion for service providers: The NSO-VFC introduces a novel feature that allows service providers to suggest suitable devices for specific services or tasks within the vehicular fog computing environment. This functionality enhances the efficiency and effectiveness of service provisioning, ensuring optimal resource utilization and improved service delivery.

Overall, the NSO-VFC scheme brings a comprehensive set of security enhancements and innovative features to the realm of vehicular fog computing, addressing key challenges and providing a secure and efficient environment for vehicular communication and service delivery.

### Paper organization

The article is organized as follows: “Related work” covers related works and the system model, while “System models” discusses assumptions, the threat model, and the problem statement. “NSO-VFC scheme” outlines the phases of the NSO-VFC scheme. In “Security analysis”, a comprehensive security analysis of the NSO-VFC is presented both formally and informally. “Performance analysis” delves into the Performance analysis of the NSO-VFC, focusing on communication, computation, costs, and security features. “Simulation” showcases the simulation results of the NSO-VFC using NS3. Finally, the conclusion is presented to summarize the findings of the study.

## Related work

In this section, the vulnerabilities and scope of existing literature are analyzed, culminating in a summary of the research studies and identification of research gaps presented in Table 1. In 2019, Tassi et al. presented a safe offloading strategy for vehicles^19^. The objective of this research was to safeguard the information exchanged between the sender and receiver by employing a random linear programming method. Nevertheless, this strategy does not propose a method for identifying the sender and receiver. In 2020, Mu et al. presented a blockchain-based and secure learning-based scheme for intelligent offloading in fog-based vehicles^20^. This approach utilizes public key and digital signature algorithms for authentication. While the blockchain system ensures security, it also introduces complexity that escalates with transaction volume. Environments like the Internet of Things and mobile devices, which possess limited processing capabilities and energy resources, may struggle to perform such intensive computations. In 2020, Iqbal et al. proposed a blockchain-based city management plan to offload low-level tasks to fog-based vehicles^21^. This strategy involves intensive computations because of its reliance on the blockchain system, making it unsuitable for environments with limited processing power. Darhab et al. In 2020, presented a mutual authentication for offloading in the mobile cloud computing^22^. The design was founded on public key cryptography and hash functions. Nevertheless, the suggested approach is susceptible to passive security attacks. Monyol et al. In 2020, proposed a secure authentication scheme for offloading services in mobile cloud computing^23^. The method provided mutual authentication between mobile and server; the scheme is based on hash functions and public keys. However, the method is vulnerable to rainbow attacks and brute force attacks. In 2021, Lakhan et al. method a motion-aware offloading scheme using the blockchain system^24^. This scheme aims to minimize the load shedding cost for mobile nodes. The proposed scheme uses hash and digital signature functions to authenticate the sender and receiver. However, the proposed system is However, it is vulnerable to passive attacks. Also, the blockchain system has many transactions, and therefore the blockchain system is not suitable for environments with limited processing power. SO for mobile edge computing using multi-vehicle multi-task non-orthogonal multiple access in the IoV was proposed in 2022 by Ling et al.^25^. The proposed scheme focuses on eavesdropping. However, it is vulnerable to passive attacks. SO for the IoV in edge computing in 2021 is proposed by Xiaolong et al.^26^. This method primarily emphasizes offloading vehicle load using Software-Defined Networking (SDN). While it can accommodate fog computing, security concerns are not addressed in this approach. Peng et al. Advanced Service Offloading Strategy for IoV in Mobile Edge Computing is suggested in 2023^27^. However, this scheme is vulnerable to both active and passive attacks and lacks support for SO. Almuseelem proposed an energy-efficient and security-conscious computing system in 2023^28^. This approach utilizes cloud and fog computing for offloading but does not address security concerns.

**Table 1:** Compares the related works.

Related work	Cloud	Fog	Mutual authentication	Key exchange	Secure	Offloading
^19^	No	No	No	No	No	No
^20^	No	Yes	Yes	No	No	No
^21^	No	Yes	Yes	No	No	No
^22^	Yes	No	No	Yes	No	No
^23^	Yes	No	No	Yes	No	No
^24^	Yes	Yes	Yes	No	No	No
^25^	Yes	Yes	No	No	No	Yes
^26^	Yes	Yes	No	No	No	Yes
^27^	Yes	Yes	No	No	No	Yes
^28^	Yes	Yes	No	No	No	Yes
NSO-VFC	Yes	Yes	Yes	Yes	Yes	Yes

Given the importance of offloading in cloud environments, security risks are inevitable in this domain. Therefore, Security SO in Fog-Cloud Federation-Based IoV stands out as a critical challenge in this field.

## System models

This section provides an overview of the network system utilized, including the threat model, followed by an explanation of the assumptions and problem statement specific to the defined network.

### Network system

**Figure 1:**
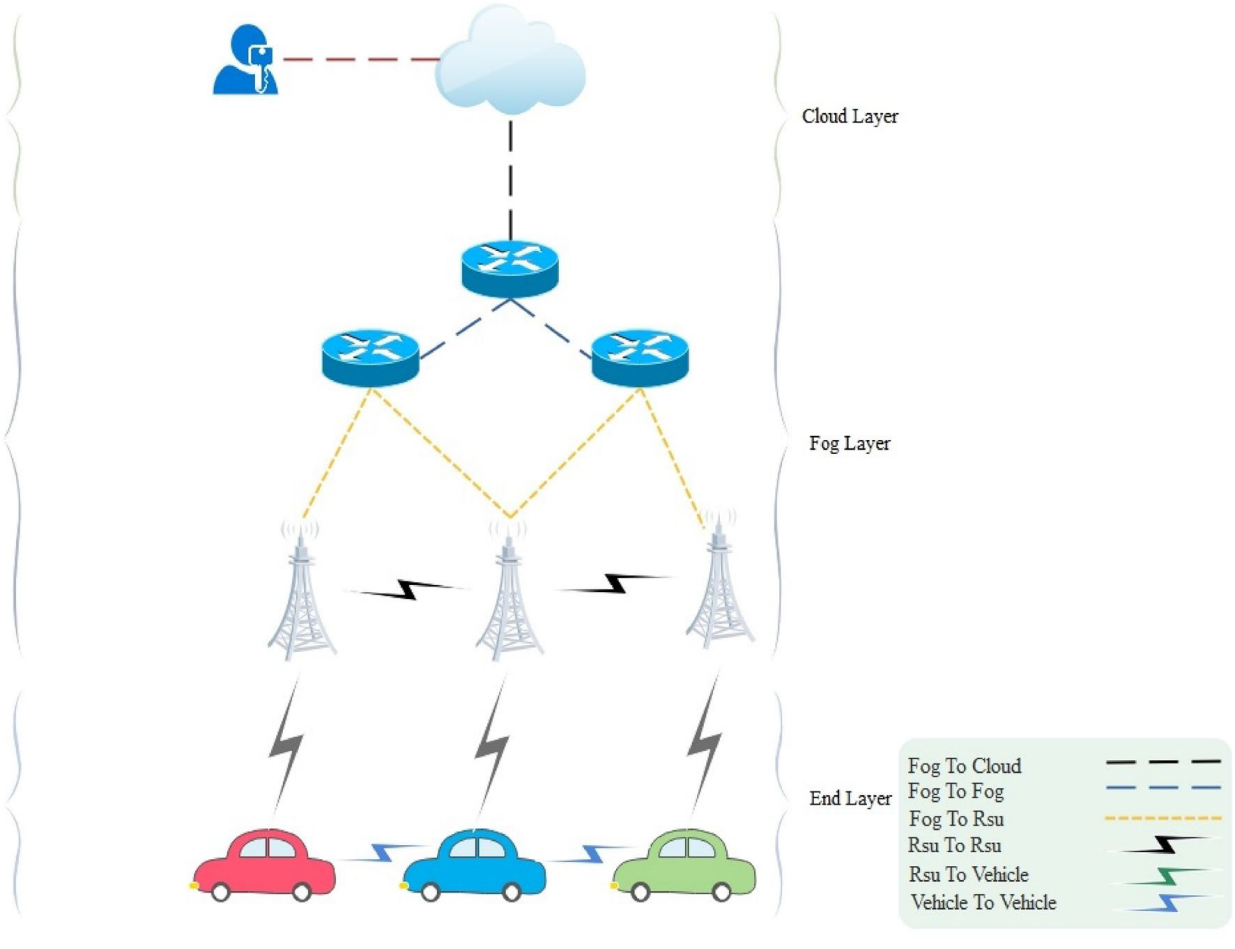
Network system.

The NSOS-VFC scheme is a sophisticated network system that is designed to efficiently handle communication and processing tasks within a hierarchical structure. At the core of this system are cloud servers, which are strategically placed in the first layer. These servers are centralized and possess high processing power, making them ideal for handling complex tasks. They are able to communicate with the trusted authority and the lower layers of the network. The second layer of the system is comprised of fog nodes, which are distributed throughout the network. These nodes have less processing power compared to the cloud servers, but they are able to manage communication within their own layer and with the upper and lower layers. The fog nodes are coordinated by a fog center, which ensures efficient communication and task distribution among the nodes. The third layer of the network system is where the end devices, known as IoVs, are located. These devices have limited processing power and rely on the fog layer for assistance with complex tasks. If an IoV is unable to handle a process due to its processing limitations, it can offload the task to the fog layer, which will then take care of processing it. Overall, the NSOS-VFC scheme is a well-structured network system that leverages the strengths of cloud servers, fog nodes, and IoVs to ensure efficient communication and processing capabilities. This hierarchical approach allows for seamless coordination and task distribution within the network. Figure 1 shows the network system.

### System threat model

In our network system, we introduce the Dolev-Yao (DY) representative attack model, which is utilized within the scheme of the NSO-VFC scheme. As per the assumptions outlined in “System assumption”, the communication channels within the network system are deemed insecure. This vulnerability opens up avenues for attackers to intercept, eavesdrop on, and manipulate messages exchanged between IoV and RSU entities. The attacker may also compromise both public and session keys utilized in the communication process. Within this attack model, all network connections are susceptible to exploitation by malicious actors. Mitigating eavesdropping and safeguarding against attacker interference present significant challenges in ensuring the security objectives of the NSO-VFC scheme. The attack model encompasses various threats, including Replay attacks, Man-in-the-middle (MITM) attacks, guessing attacks, brute-force attacks, Sniffer attacks, Impersonation attacks, Rainbow table attacks, and Stolen verifier attacks, all of which have the potential to compromise the security of the NSO-VFC scheme.

### System assumption

- The NSOS-VFC scheme operates under the assumption that a malicious entity can impersonate any communicating party at any point during the communication process.
- There is a possibility of active and passive attacks in the stage of offloading.
- It is assumed that the attacker has high processing power and can perform attacks such as rainbow and brute force.
- Communication channels within the network are deemed insecure, leaving them vulnerable to interception and manipulation.
- There are no corrupt or rogue nodes that send data to the attacker.
- IoVs are unaware of the identities of access points, fog nodes, and cloud servers.
- Mutual awareness exists between the fog layer and cloud servers.
- Cloud servers are safeguarded against vulnerabilities.
- Data is not lost in cloud servers.
- The fog layer and access points have mutual knowledge of each other's identities.
- Each fog knows its subset of fog nodes.
- Data stored within the fog layer is secure and remains uncompromised.
- RSUs cannot alone act as representative devices for task offloading.
- The fog center can make decisions regarding device selection for offloading based on processing power.
- The fog center possesses information regarding the energy status of all IoVs.
- The device to be proposed for offloading has already been authenticated.
- Cloud servers must be informed by the fog center when a device is selected for offloading.
- Due to limited memory in RSUs, all encryption keys are centrally stored within the fog center.
- All IoVs within the fog and cloud environments operate on synchronized time settings.

### Problem statement

IoVs, being mobile entities, are inherently constrained by limitations in memory and processing power. To address the challenges posed by heavy processing tasks, IoVs employ offloading strategies to offload tasks to external resources. However, the reliance on offloading introduces security vulnerabilities within the fog-cloud federation environment. Previous research often assumed a level of trust among vehicles in the fog environment, neglecting the potential threats associated with offloading. In this study, we aim to bridge the gap between theoretical assumptions and real-world scenarios by acknowledging the presence of security threats such as MITM attacks, replay attacks, and forgery attacks in our environment. It is imperative for IoVs to establish mutual trust and verify each other's identities before engaging in offloading tasks within the fog-cloud federation. Developing a robust authentication mechanism capable of securely confirming the identities of communication parties and resilient against both active and passive attacks is paramount in ensuring the integrity and security of the system.

## NSO-VFC scheme

**Figure 2:**
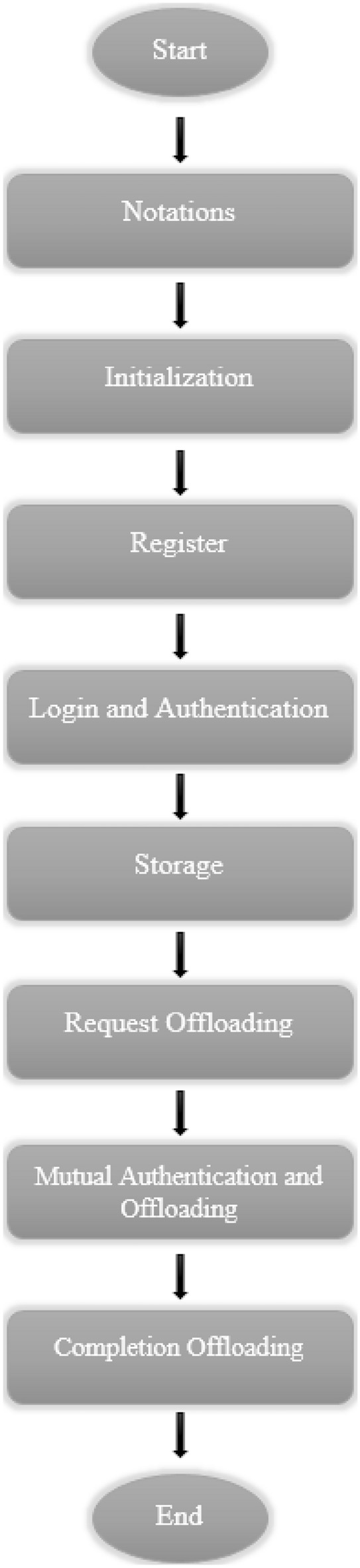
Roadmap of the NSO-VFC.

This section organizes the NSO-VFC into nine steps for description. Figure 2 shows the roadmap of the NSO-VFC.

### Notations

Table 2 shows the Notations used in the NSO-VFC.

**Table 2:** Notations used in the NSOS-VFC.

Notation	Description
Cs	Cloud computing
F	Fog computing
R	Rsu (fog node)
Vi	IoV
TA	Trusted authority
Id_Cs_	Identity of Cs
Id_Fi_	Identity of Fi
Id_Ri_	Identity of Ri
Idvi	Identity of Vi
Id_TA_	Identity of TA
Id_Visf_	Identity suggested IOV to offload
r_1_, r_2_, r_3_, r_4_	Random number for generation Ecc
R1	Random number generation by IdRi
R2	Random number generation by Id_Fi_
Pow	IoV processing power
Pr	Processing requirements
Pwvi	Password of Idvi
NFR	Number of offloading requests Idvi
SFIdVisf	Successful offloading score IdVisf
BuIdVisf	Busy IdVisf. It can be true or false
SKVR	The session key in Login and authentication phase
Skof	The session key in offloading phase
T_Cs_	Timestamp Cs
T_Fi_	Timestamp R
T_Ri_	Timestamp F
TVi	Timestamp Vi
TVi_sf_	Timestamp IdVisf
∆T	Maximum message transmission delay
h (·)	Hash function
⊕	XOR
||	Concatenation operation

### Initialization phase

**Figure 3:**
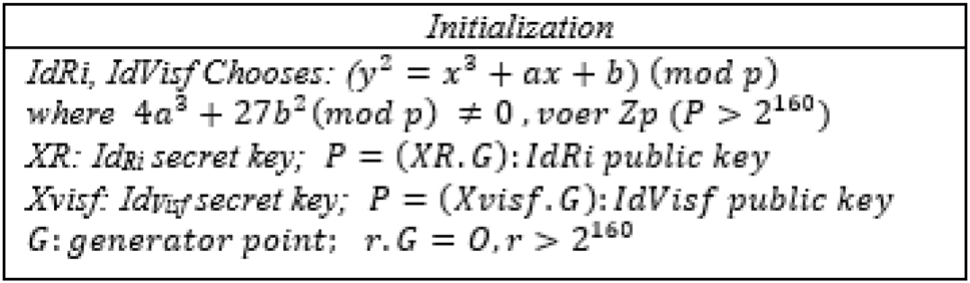
Initialization of the NSO-VFC.

The first, IdRi and IdVisf, select the elements aRi, bRi, aVisf, bVisf, and ∈ zp and satisfy the condition (${{\text{y}}}^{2}={{\text{x}}}^{3}+{\text{ax}}+{\text{b}}$) (mod p) on the elliptic curve. In an elliptic curve, *G* is the base point, with the first order of *n* (*n* > ${2}^{160}$). XR and Xvisf are randomly chosen as secret keys of IdRi and IdIisf. The public key can be generated by multiplying the secret key in G. Figure 3 shows the initialization phase.

### Register phase

Step 1: In the registration phase, Idvi first computes Pi = h (Idvi || TVi), Ps = h (Pwvi ⊕ Pi), Pw = h (Pow ⊕ Pi), Psw = h (((Ps|| Pw) ⊕ Pi) ⊕ TVi) and sends {Idvi, TVi, Psw, Pi, Ps, Pw,} to IdRi.

Step 2: After receiving the message from Idvi, IdRi It checks the time stamp, if it is smaller, it computes Pi′ = h (Idvi||TVi) and if Pi = Pi′, computes Pw′ = h (Pow ⊕ Pi), if Pw = Pw′, Psw′ = h (((Ps||Pw) ⊕ Pi) ⊕ TVi), if Psw = Psw′ Computing HRr = h (((IdRi||IdFi || TRi) ⊕ Ps) ⊕ Psw), Rr = (((IdRi||IdFi||TRi) ⊕ Ps) ⊕ Psw), PK = h (((IdRi ⊕ XR)||(IdRi ⊕ IdFi) ⊕ R1) ⊕ (Ps ⊕ Psw))), Idvi′ = PK. G, Pkr = h (IdRi|| HRr|| Idvi′), Pkr* = Pkr.G, HRPr* = h (Rr, Idvi′, HRr, TRi) and Storage Pkr*, Idvi′, ps, Pow, in memory and then Send {Rr, Idvi′, HRr, HRPr*, TRi} to the Idvi.

**Figure 4:**
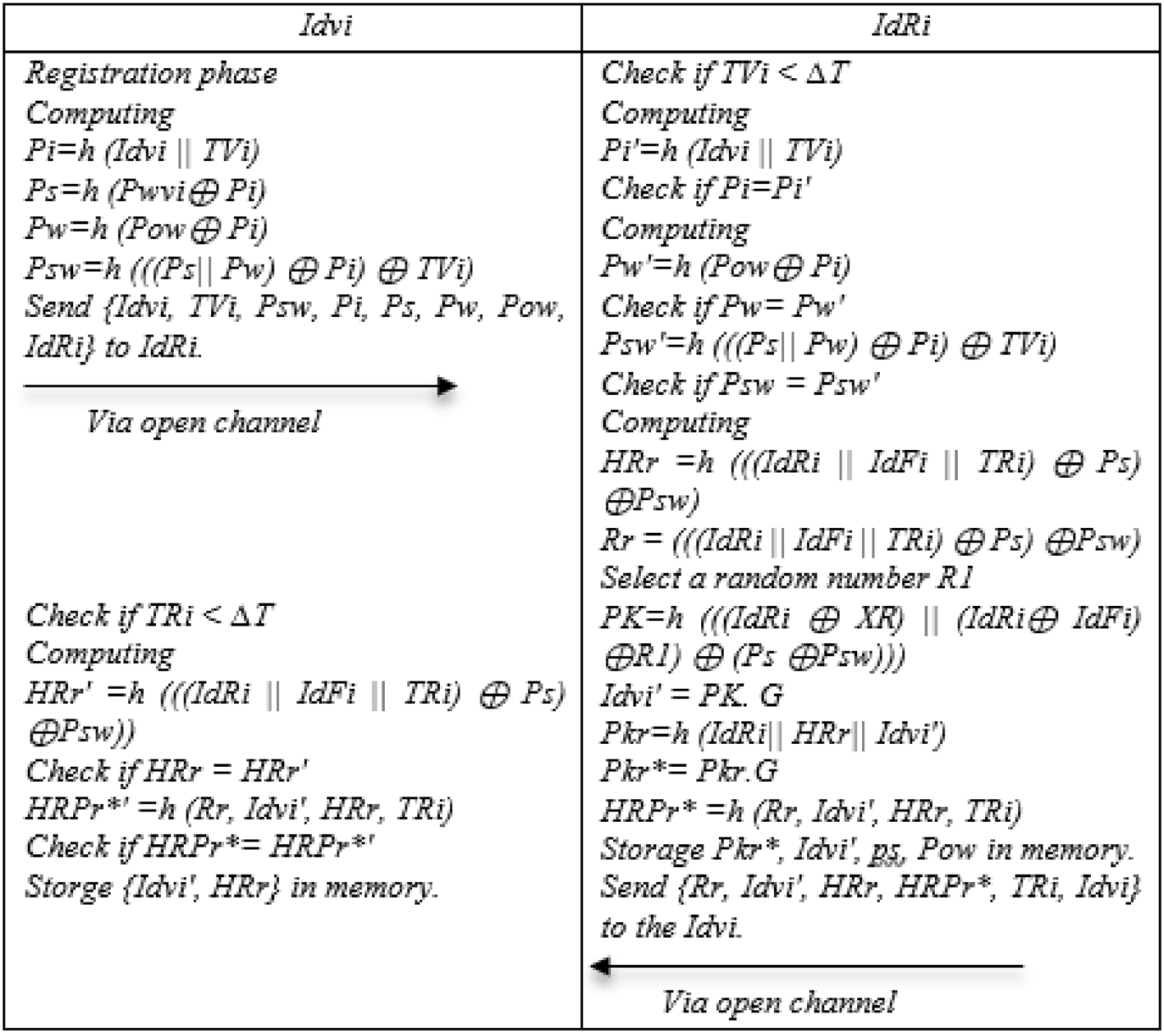
Registration of the NSOS-VFC.

Step 3: Upon receiving the message from IdRi side, Idvi first checks the time stamp of the package, if it is smaller than the expiration time, it computes HRr′ = h (((IdRi||IdFi||TRi) ⊕ Ps) ⊕ Psw)), if HRr = HRr′ computes HRPr*′ = h (Rr, Idvi′, HRr, TRi) and if HRPr* = HRPr*′, Storge {Idvi′, HRr} in memory. Figure 4 shows the registering phase.

### Login and authentication phase

Step 1: To Login and authentication, Idvi chooses a random number r1 and computes Pvar1 = (r1.G), Pvar2 = h (r1.Idvi′), Pvar3 = h((Pvar 1 ⊕ Pvar 2 ⊕ TVi)||(IdRi ⊕ Idvi) || HRr) and {Pvar1, Pvar2, Pvar3, TVi, Idvi, Idvi′, IdRi, HRr} to IdRi.

Step 2: Upon receiving the message from Idvi side, IdRi first checks the time stamp, if it is smaller than the expiration time, it computes HRr′ = h (((IdRi||IdFi||TRi) ⊕ Ps) ⊕ Psw)) if HRr = HRr′, Pvar3′ = h ((Pvar1 ⊕ Pvar2 ⊕ TVi)||(IdRi ⊕ Idvi)||HRr), Check Pvar3 = Pvar3′ Idvi′ = Idvi*′ Pvar2* = h (Pvar1. Idvi′) and Verify Pvar2* = Pvar2. If true, then select a random number r2 and computing Pvar4 = r2.G; Pvar5 = r2. Pkr*, Pvar6 = h ((Pvar4 ⊕ Pvar5)||((IdRi ⊕ TRi)||Idvi′)) and Send {Pvar4, Pvar5, Pvar6, IdRi, TRi} to Idvi.

Step 3: Idvi first checks the time stamp, if it is smaller than the expiration time, it computes Pvar6′ = h ((Pvar4 ⊕ Pvar5)||((IdRi ⊕ TRi)||Idvi′)), if Pvar6 = Pvar6′, Pkr = h (IdRi||HRr||Idvi′).

Pkr* = Pkr.G, Pvar5* = Pvar4. Pkr* and Verify Pvar5* = Pvar5. In the following Computing SKVR = r1 Pvar7 = r1.r2.G; VAR = h ((r1 Idvi′ ||SKVR)), Vh = h ((VAR ⊕ Ps ⊕ Pow ⊕ TVi|(IdRi ⊕ Idvi ⊕ HRr)), Send {VAR, Vh, Ps, Pow, TVi, IdRi, Idvi, HRr} to IdRi.

Step 4: First, IdRi checks the time stamp of the received data and if it is smaller than the expiration time, it calculates Vh′ = h ((VAR ⊕ Ps ⊕ Pow ⊕ TVi)||(IdRi ⊕ Idvi ⊕ HRr)). If it is equal Vh = Vh′, it computes SKVR* = r2.Pvar8 = r2.R1.G; VAR* = h ((Pvar8.Idvi′)||SKVR*)), Verify VAR* = VAR If true, Rsu authentication IoV; Otherwise, VAR rejected. After mutual authentication, session key SKVR = r1. Pvar7 = r1. r2. G = r2. Pvar8 = SKVR * is shared. Storage Pow in memory.vi = h ((Idvi′ ⊕ Idvi)||vp = h (Ps ⊕ Pow ⊕ TRi)||vrf = h (IdRi ⊕ IdFi)) Rv = h ((vi||vp)||vrf)) Send {Idvi′, Idvi, Pow, Ps, TRi, IdRi, IdFi, vi, vp, vrf Rv} to IdFi.

**Figure 5:**
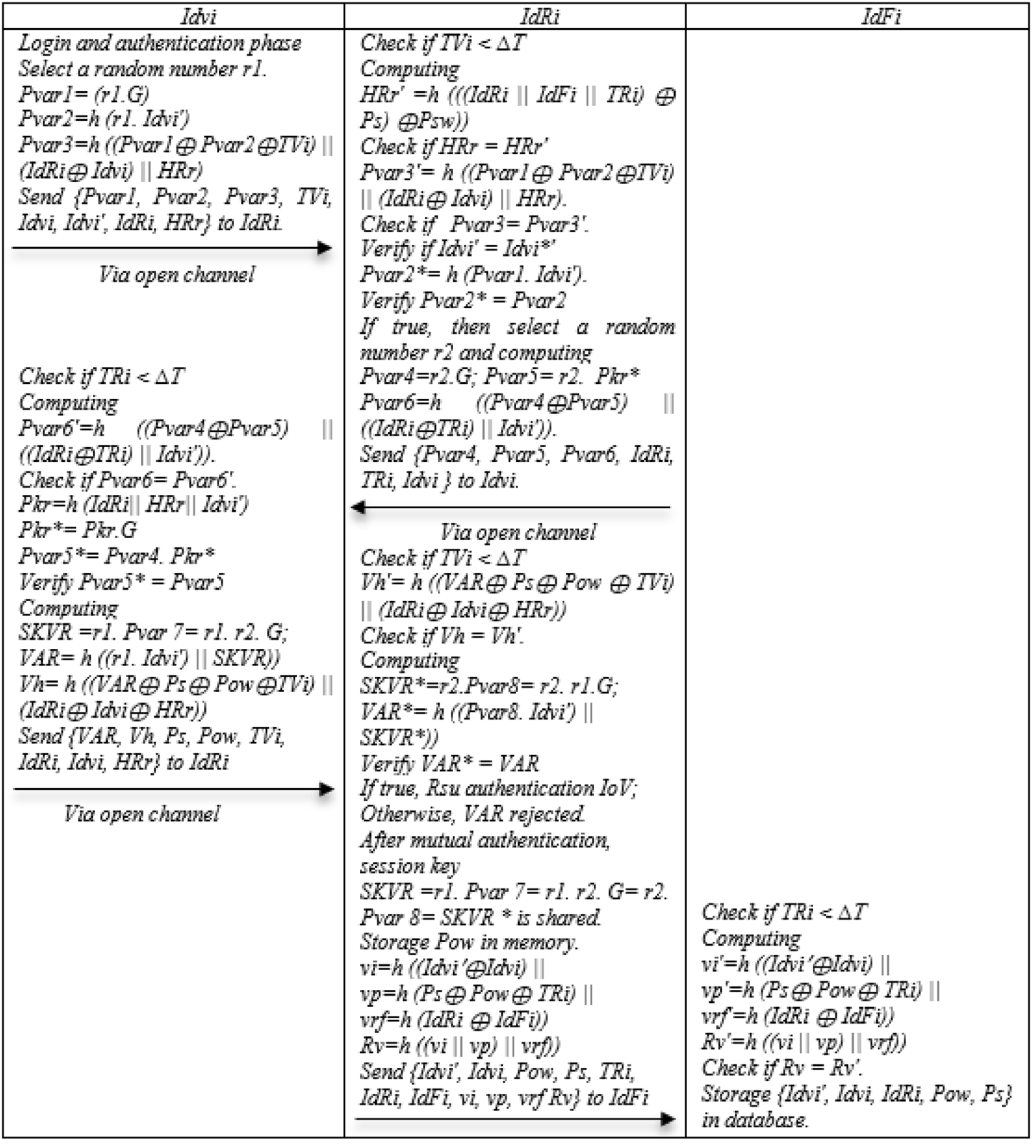
Login and authentication of the NSOS-VFC.

Step 5: First, IdFi checks the time stamp for the messages received from IdRi side. If the time stamp is smaller than the expiration time, he Computing vi′ = h ((Idvi′ ⊕ Idvi)||vp′ = h (Ps ⊕ Pow ⊕ TRi)||vrf′ = h (IdRi ⊕ IdFi)), Rv′ = h ((vi||vp)||vrf)), Check if Rv = Rv′. Storage {Idvi′, Idvi, IdRi, Pow, Ps} in database. Figure 5 Shows Login and authentication phase.

### Storage phase

Step 1: IdFi Computing iv = (Idvi′ ⊕ Idvi), pv = (Ps ⊕ Pow), Fvi = ((IdFi ⊕ iv)||(IdRi ⊕ pv)), HFvi = h ((Fvi ⊕ IdCs)||TFi)) and Send {iv, pv, HFvi, Fvi, IdFi, TFi, IdRi, IdCs} to IdCs.

Step 2: First, IdCs checks the time stamp of the received data and if it is smaller than the expiration time, it Computing Fvi = ((IdFi ⊕ iv)||(IdRi ⊕ pv)), HFvi′ = h ((Fvi ⊕ IdCs)||TFi)), if HFvi = HFvi′ Computing CFi = ((IdCs ⊕ Fvi)||IdTA), CFi′ = h ((IdCs ⊕ Fvi)||(IdTA ⊕ TCs), Storage {CFi} in database, In the following Send {CFi′, IdFi, IdCs, TCs} to IdFi and Send {CFi, IdCs, TCs, IdTA} to IdTA.

**Figure 6:**
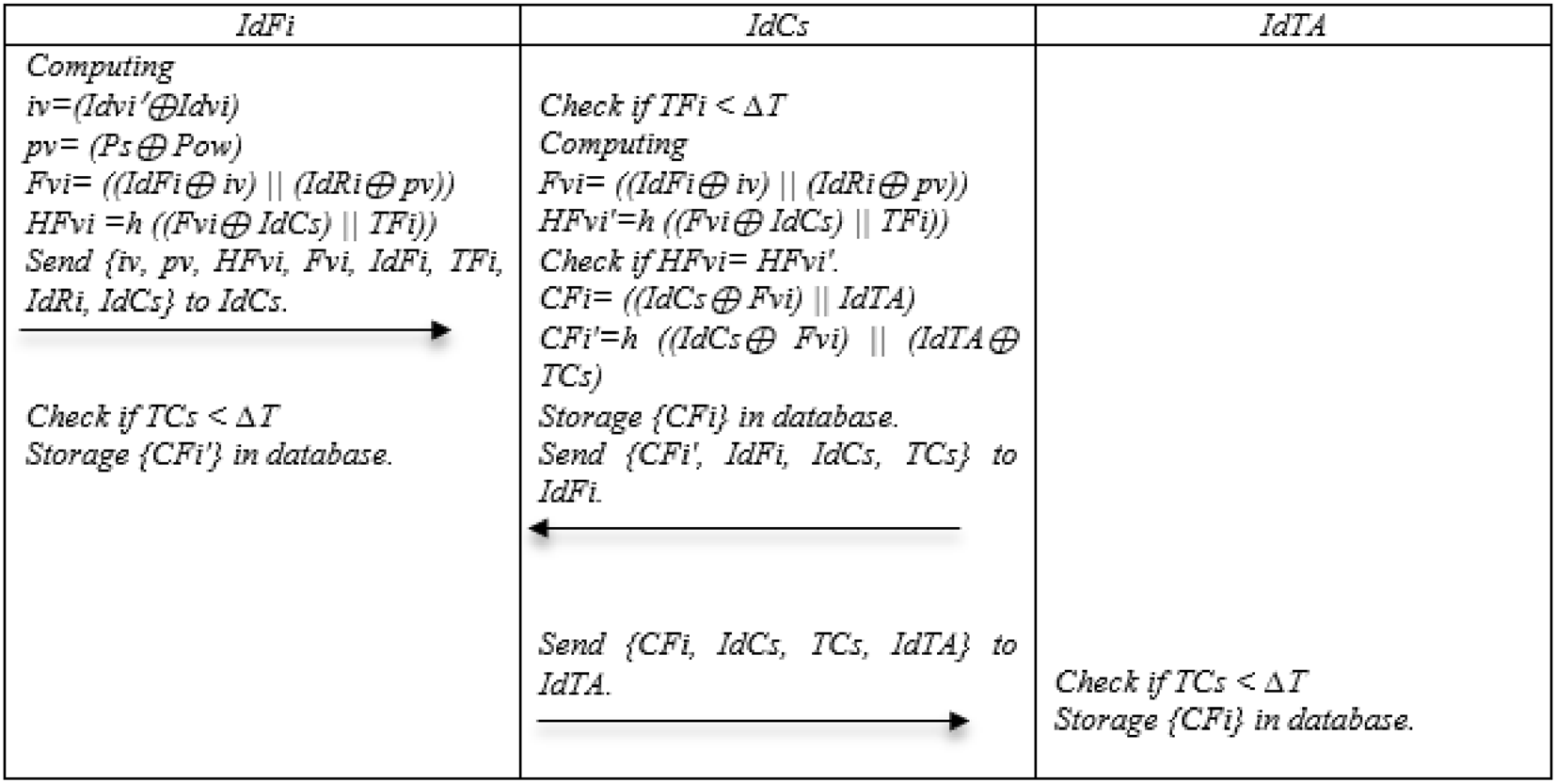
Storage of the NSOS-VFC.

Step 3: IdFi and IdTA check the time stamp; if it is smaller than the expiration time, IdFi stores CFi′ and CFi is stored by IdTA in database. Figure 6 Shows storage phase.

### Request offloading phase

Step 1: In some cases, Idvi has heavy tasks that are beyond his power and energy. In this case, he must offload his data to another device for processing. Because it does not know the processing power of nearby devices, it computes Rf = h (Idvi′ ⊕ Pr ⊕ TVi) and Send {Rf, Idvi′, Pr, TVi} to IdRi.

Step 2: IdRi receives Idvi request to offload the cargo, checks the time stamp to respond to Offloading request, and if it is smaller than the expiration time, Computing Rf′ = h (Idvi′ ⊕ Pr ⊕ TVi), if Rf = Rf′, PK = h (((IdRi ⊕ XR)||(IdRi ⊕ IdFi) ⊕ R1) ⊕ (Ps ⊕ Psw))), Idvi′* = PK. G and Verify Idvi′* = Idvi′ Send {Idvi′*, Idvi′, Pr, TRi} to IdFi.

Step 3: IdFi receives the request offloading and first checks the time stamp if it is smaller than the expiration time, Checking Idvi′ from the database if Verify VIdvi′* = Idvi′* = Idvi′, Search for required processing power. If available Idvisf′ = h ((Idvisf ⊕ Xvisf ⊕ IdFi), FR = h ((VIdvi′* ⊕ Idvi′)||(Pow, ⊕ Pr)||(Idvisf ⊕ TFi) ⊕ IdRi) ⊕ Idvisf′) and adds 1 to the value of NFR parameters and BuIdVisf = true. Send {VIdvi′*, Idvi′, Pow, Pr, Idvisf, Idvisf′, TFi, IdRi, IdFi, FRH, BuIdVisf} to IdRi and Send {CFi′} to IdCs.

Step 4: IdRi receives the response and first checks the timestamp that is less than the expiration time Computing Fr′ = h ((VIdvi′* ⊕ Idvi′)||(Pow, ⊕ Pr)||(Idvisf ⊕ TFi) ⊕ IdRi)) ⊕ Idvisf′) if Fr = Fr′.Pr′ = h (Pow ⊕ Pr ⊕ Idvisf ⊕ Idvisf′), Resf = h (Idvisf ⊕ Idvisf′ ⊕ TFi ⊕ Idvi),Storage Idvisf, Idvisf′, Pr ′ in memory and Send {Idvisf, Idvisf′, TFi, Idvi, Resf, BuIdVisf} to Idvi.

**Figure 7:**
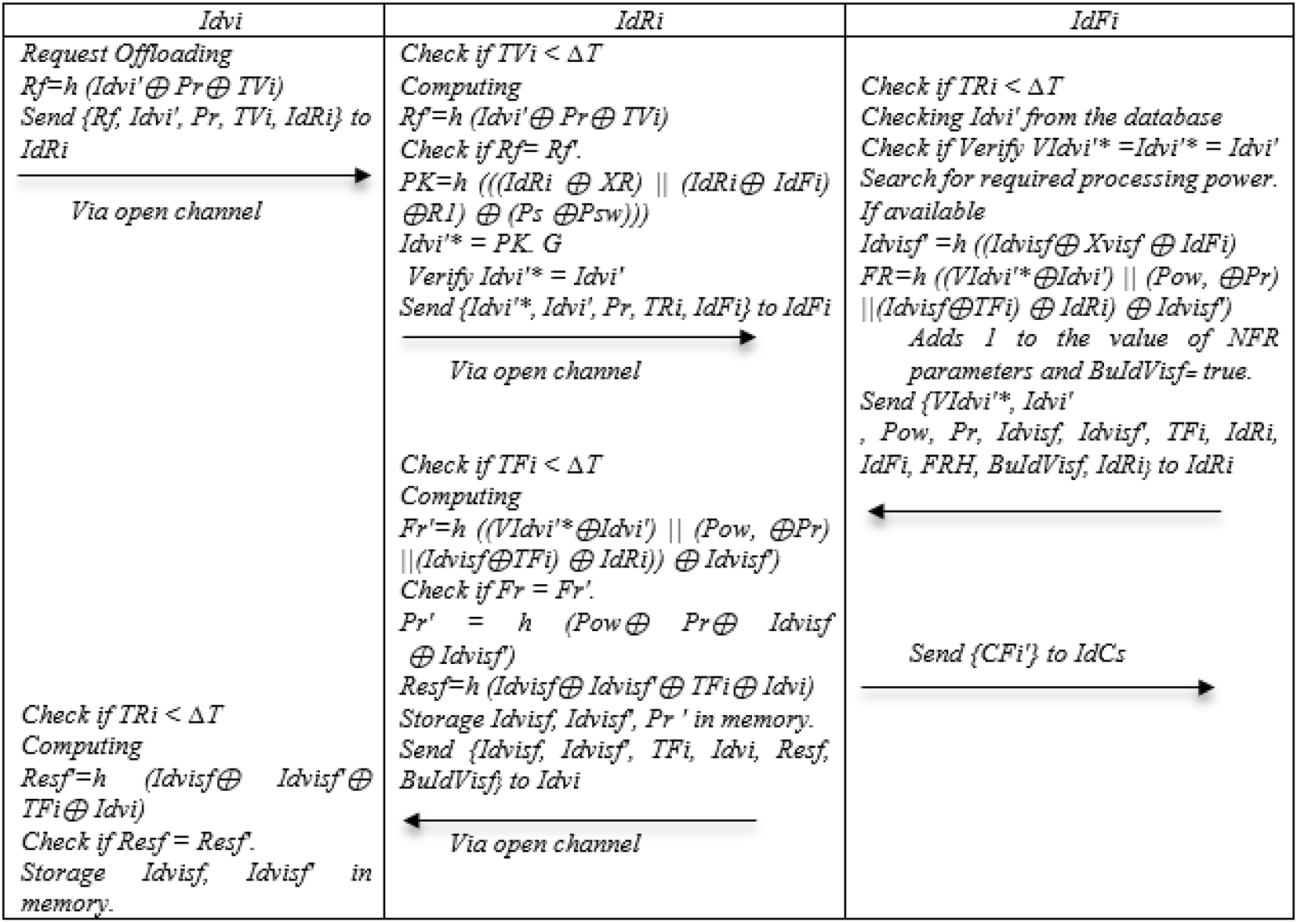
Request offloading of the NSOS-VFC.

Step 5: Idvi receives the offload proposal response and first checks the timestamp that is less than the expiration time and Computes Resf′ = h (Idvisf ⊕ Idvisf′ ⊕ TFi ⊕ Idvi) if Resf = Resf′, Storage Idvisf, Idvisf′ in memory. Figure 7 Show Request offloading phase.

### Mutual authentication and offloading phase

Step 1: Idvi for the offload request calculates Computing OFi = h (Idvi||TVi ⊕ BuIdVisf) Select a random number r3, Pofv1 = r3.G, Pofv2 = h (r3. Idvisf′), Pofv3 = h (Pofv1, Pofv2, TVi) and Send {Idvi, Idvi′, TVi, OFi, Pofv1, Pofv2, Pofv3, BuIdVisf} to Idvisf.

Step 2: Idvisf receives Idvi Offloading request and first checks the time stamp, if it is smaller than the expiration time, it Computes OFi′ = h (Idvi||TVi ⊕ BuIdVisf) if OFi = OFi′, Fisf = h ((Idvisf||TVisf) ⊕ IdFi)||BuIdVisf) and Send {Idvi′, Fisf, TVisf, Idvisf, IdRi} to IdRi.

Step 3: IdRi receives Idvisf request and first checks the timestamp, if it is less than the expiration time Computes Fisf′ = h ((Idvisf||TVisf) ⊕ IdFi)||BuIdVisf), PK = h (((IdRi ⊕ XR)||(IdRi ⊕ IdFi) ⊕ R1) ⊕ (Ps ⊕ Psw))), Idvi′* = PK. G, Verify Idvi′* = Idvi′ If true, Send {Idvi′*, Idvisf, TRi} to Idvisf.

Step 4: Idvisf receives IdRi message and first checks the timestamp, if it is less than the expiration time, Verify Idvi′* = Idvi′If true, Pofv3′ = h (Pofv1, Pofv2, TVi), if Pofv3 = Pofv3′. Obtains data Pofv2* = h (Pofv2. Idvisf′), Verify Pofv2* = P10 If true, then select a random number r4 and computing Pofv4 = r4.G, Pofv5 = h (r4. (HRr ⊕ Idvisf′) ⊕ Idvi′), Pofv6 = h ((Pofv4 ⊕ Pofv5)||(Idvi′ ⊕ TVisf) and Send {Pofv4, Pofv5, Pofv6, TVisf} to Idvi.

Step 5: Idvi first checks the timestamp, if it is less than the expiration time, computes Pofv6′ = h ((Pofv4 ⊕ Pofv5)||(Idvi′ ⊕ TVisf) if Pofv6 = Pofv6′, HRr = h (IdRi||IdFi||TRi), Pofv5* = h (Pofv4. (HRr ⊕ Idvisf′) ⊕ Idvi′), Verify Pofv5* = Pofv5, Computing Skof = r3. Pofv7 = r3. r4.G, Vof = h ((r3. Idvisf′)||Skof)) and Send {Vof, TVi, Idvisf} to Idvisf.

**Figure 8:**
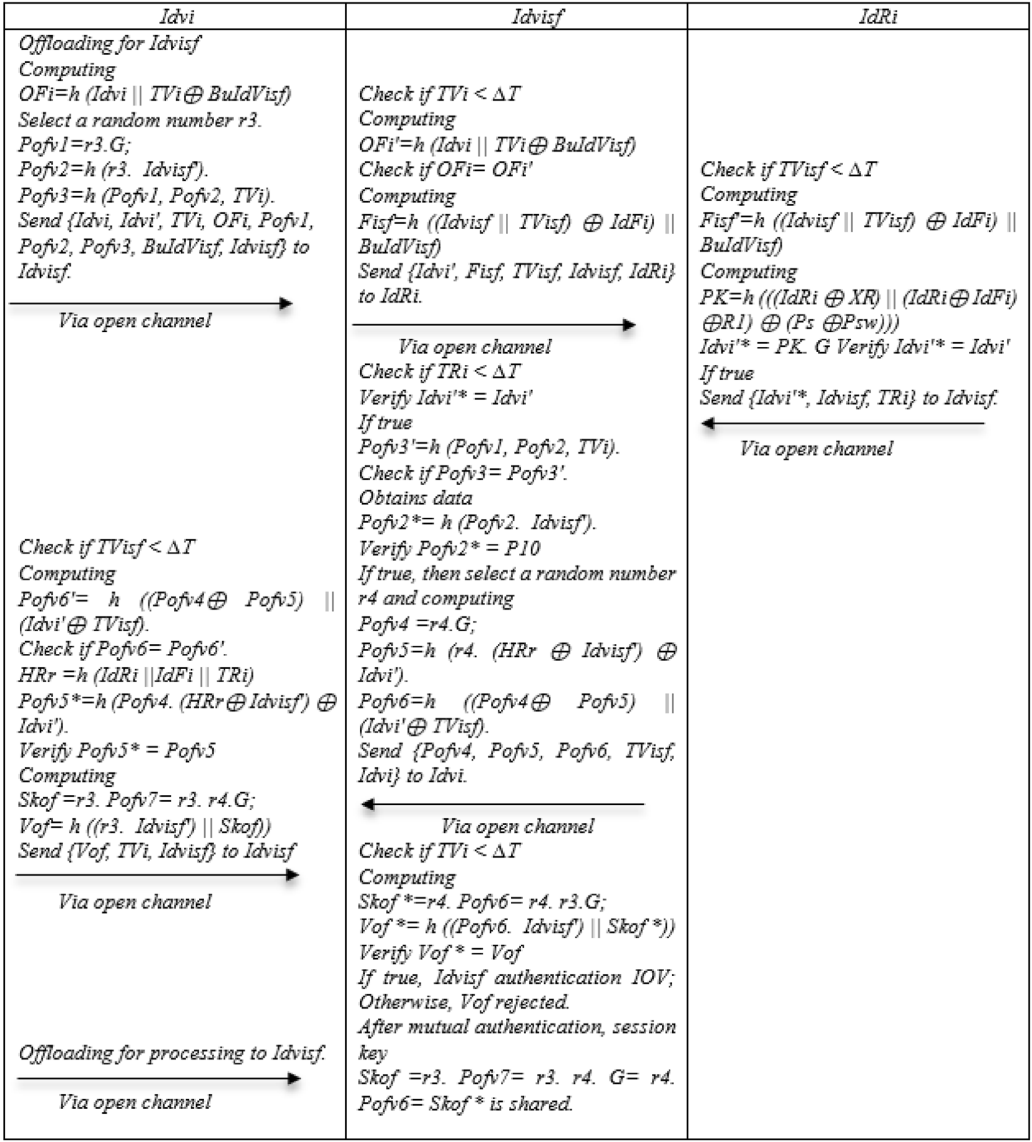
Mutual Authentication and offloading of the NSOS-VFC.

Step 6: Idvi first checks the timestamp, if it is less than the expiration time computes Skof * = r4. Pofv6 = r4. r3.G; Vof * = h ((Pofv6. Idvisf′)||Skof *)), Verify Vof * = Vof, if true, Idvisf authentication IoV; Otherwise, Vof rejected., After mutual authentication, session key, Skof = r3. Pofv7 = r3. r4. G = r4. Pofv6 = Skof * is shared. After this stage, Idvi can offload the process. Figure 8 shows Mutual Authentication and offloading phase.

### Completion of the offloading phase

Step 1: After authenticating and receiving the offloading for processing from Idvi, it starts processing and after finishing the processing, it sends {Processing result, IdIi, IdIisf} to Idvi and {Completion offloading, IdIisf, IdRi, BuIdVisf} to IdRi.

Step 2: Idvi, who received the result of offloading, sends {Completion offloading, Idvi IdIisf, IdRi, BuIdVisf} to IdRi.

Step 3: After receiving the message from IdIisf and Idvi, IdRi sends {Completion offloading, IdIisf, IdRi, IdFi, BuIdVisf} to IdFi, which indicates that the offloading has been done and the result has been produced.

**Figure 9:**
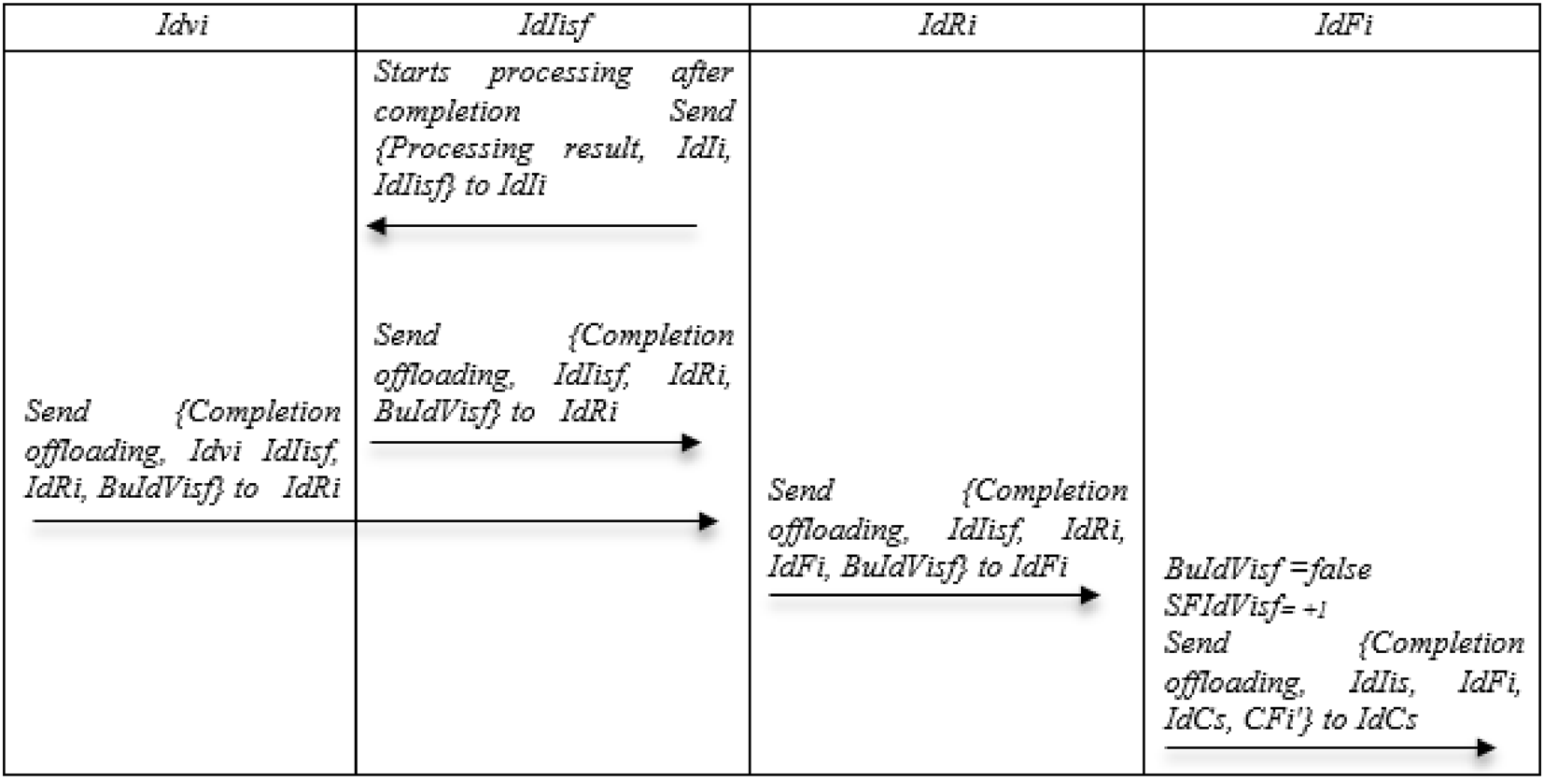
Completion of the offloading of the NSOS-VFC.

Step 4: Upon receiving the message, IdFi does BuIdVisf = false and SFIdVisf =  + 1and sends {Completion offloading, IdIis, IdFi, IdCs, CFi′}to IdCs. Figure 9 show Completion of the offloading phase.

## Security analysis

This section presents an informal and formal security analysis of the NSO-VFC.

### Informal analysis

This section presents an informal analysis of the attacks defined as threats to the NSO-VFC in “System threat model”.

#### Replay attack

In this attack, the main goal of the attacker is to record the exchanged message in the network so that he can re-inject the recorded messages in the network after some time. In NSO-VFC, the attacker may want to capture the messages exchanged in all phases and use it as a legal tool to intrude. In the NSO-VFC, the time stamp TCs, TFi, TRi, TVi and TVisf is defined for the roles Cs, F, R, Vi, and in all exchanged messages, the time stamp is checked first before each action; if it is smaller than ∆T, the relevant operation is performed.

#### MITM attack

In this attack, the attacker is placed between two communication parties, the main goal of the attacker is to control the communication between the communication parties. In NSO-VFC mutual authentication is done between Idvi, IdRi and Idvi, IdVisf which disables MITM attack. Sections “Login and authentication phase” and “Mutual authentication and offloading phase” show the steps of mutual authentication.

#### Brute-force attack

In this attack, the attacker checks all the possible state space to break the public or private keys used in the communication. To attack NSO-VFC, the attacker must extract all parameters of (Pvar1, Pvar2, Pvar3, Pvar4, Pvar5, Pvar6, Pvar7, Pvar8) and (Pofv1 Pofv2 Pofv3 Pofv4 Pofv5 Pofv6, Pofv7, Pofv8) from the messages, which is practically impossible due to checking ∆T; however, if the attacker can extract these parameters, he cannot do non-virtual work because the keys of XR and Xvisf is unknown to him, and there is nothing for him to randomly sense the numbers r1, r2, r3, r4, R1, and R2.

#### Impersonation attack

In this attack, the attacker monitors the legal messages sent in the network and tries to use the contents of the monitored messages for valid requests in the following sessions to gain illegal access. Assuming that the attacker wants to falsify the identity of Idvi, since the values are in memory and it is impossible to access them correctly, and that the attacker cannot forge the identity of Idvi without knowing the points of Pvar1 and Pvar2 and the secret key of PK = h (((IdRi ⊕ XR)||(IdRi ⊕ IdFi) ⊕ R1) ⊕ (Ps ⊕ Psw))).

#### Guessing attack

In this attack, the attacker aims to guess the password offline and online. Considering that Idvi calculates Pi = h (Idvi||TVi), Ps = h (Pwvi ⊕ Pi), Pw = h (Pow ⊕ Pi), Psw = h (((Ps||Pw) ⊕ Pi) ⊕ TVi), at the beginning and sends it to IdRi, no one except Idvi knows the contents of the transmitted data. Offline and online solutions are impossible without knowing the identity and password, so the NSO-VFC resists guessing attack.

#### Session key leakage attack

After the authentication, Idvi, IdRi and Idvi, IdVisf will share the SKVR = r1. Pvar 7 = r1. r2. G = r2, Skof = r3. Pofv7 = r3. r4. G = r4. session keys. r1, r2, r3, r4 random numbers generate the session key information of the communication parties, so the attacker cannot calculate the session key. The NSO-VFC guarantees the confidentiality of the session key.

#### Stolen verifier attack

In this attack, the attacker tries to attack the database to steal the stored usernames and passwords and use them for legitimate access. In the NSO-VFC, it is assumed that the fog layer and the cloud are not vulnerable; however, if the attacker can access the information stored in the cloud, a secret key and guessing random numbers are needed to calculate the CFi = ((IdCs ⊕ Fvi)||IdTA), CFi′ = h ((IdCs ⊕ Fvi)||(IdTA ⊕ TCs), with the CFi in hand, the attacker cannot Create a legitimate request for authentication.

#### Rainbow table attack

In this attack, the attacker uses a pre-computed database to break hash functions. The NSO-VFC is used in all stages of sending and receiving timestamped messages. If the time stamp of any sent packets is greater than the expiration time, the next step will not be performed, so the NSO-VFC is resistant to Rainbow table attack.

#### Sniffer attack

In this attack, the attacker tries to access the confidential data of the correct users by commenting and monitoring the network. Then she can establish legal communication between the parties. In the NSO-VFC, the messages sent between the communication parties are sent in the form of a hash; for this reason, the attacker cannot monitor the security parameters.

#### Provides confidentiality

In this, we used the hash function and ECC to SO for IoV. In this scheme, only authorized devices are allowed legal access. The examined items from “Replay attack” to “Sniffer attack” show that the proposed scheme is resistant to known attacks.

### Formal analysis

This section provides a formal security analysis with Avispa for NSO-VFC.

#### AVISPA

AVISPA tool is a tool for formal security evaluation of protocols, and the simulation results show the safe or unsafe security of the protocol^29^. First, the protocol is written in HLPSL language to evaluate the security, HLPSL is a role-based language^30^. Each role is independent of other roles, and communication between roles is possible through channels. In the HLPSL language, the attacker is modeled by the DY model in this tool^31^. First, HLPSL codes are converted into IF by the hlpsl2if translator and then executed as AVISPA input; IF is a low-level language compared to the HLPSL language, and the translation of HLPSL codes to IF is hidden from the user^32^. Avispa has four backends, (OFMC)^33^, a (CL-AtSe)^34^, (SATMC)^35^, (TA4SP)^36^, Can be used to test whether a protocol is secure or insecure.

#### Simulation results

**Figure 10:**
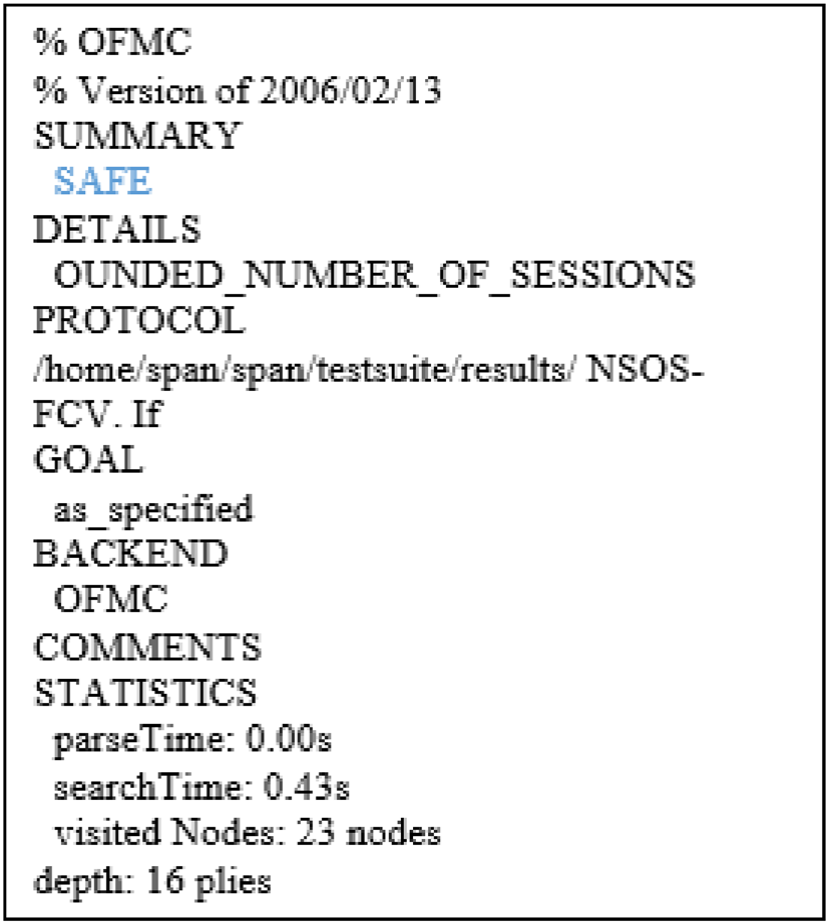
Security results of OFMC for NSO-VFC.

**Figure 11:**
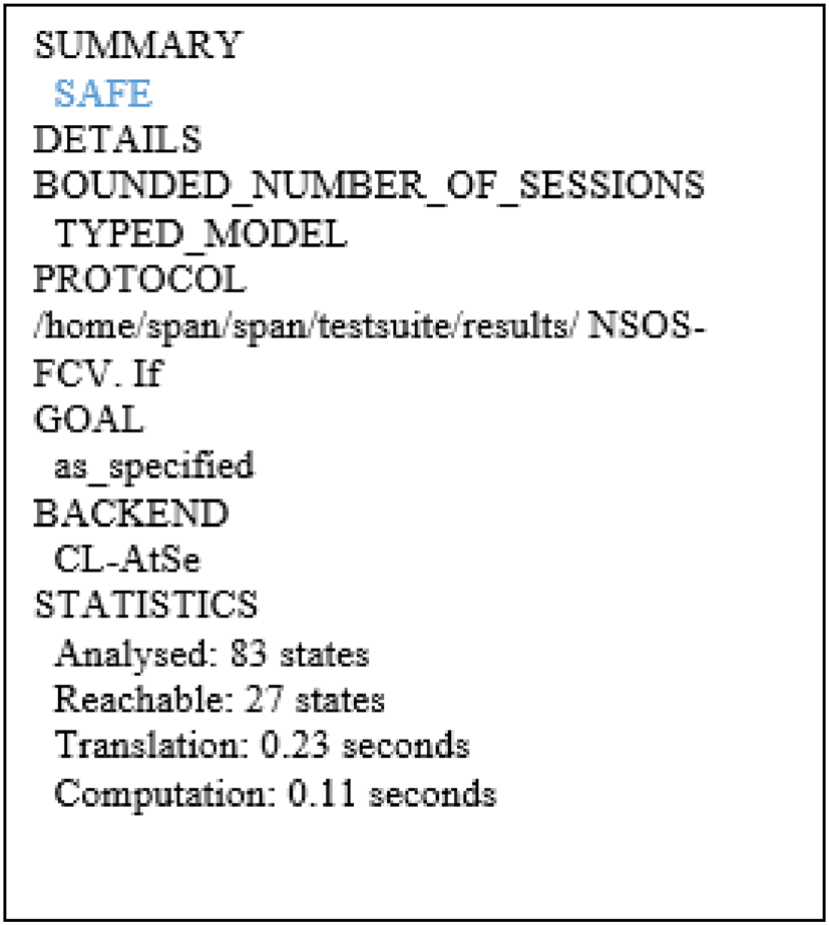
Security results of CL-AtSe for NSO-VFC.

Tool Avispa gives us three mechanisms to check the scheme proposal. In the first mechanism, the replay attack is investigated in the DY model, and the second mechanism is designed with the protocol specifications and activates intruder recognition. The third mechanism examines the MITM attack. The NSO-VFC has been investigated with three mechanisms defined in the Avispa tool. The results show that it resists repetition attacks, active intruders, and MITM attacks. Figures 10 and 11 show the output results of OFMC and CL-AtSe.

## Performance analysis

The proposed of NSO-VFC consists of 7 phases; in the first phase, considering that the devices have a pre-agreement on the elliptic equations, it is not considered in the performance phase (this is a fair assumption that is applied in other similar methods produced has been. We have divided the remaining 6 phases into six situations to analyze and read the performance, followed by the Communication cost and the number of bits and the analysis of the Computation cost. In the end, the Security requirements are expressed in this section.

- state 1: Register phase.
- state 2: Login and authentication phase.
- state 3: Storage phase.
- state 4: Request offloading phase.
- state 5: Mutual Authentication and offloading phase.
- state 6: Completion of the offloading phase.

Manual methods are used in all articles to calculate the communication and Computation cost, which is a possibility of human error. We used E3C to reduce human error in calculating Computation and communication costs^37^. The combined manual and E3C methods results are in “Communication cost” and “Computation cost”.

### Communication cost

To calculate the communication cost in the NSO-VFC, we considered the 160-bit integers, the hash function of the 160-bit SHA1, and the elliptic curve encryption type with a key size of 160. In other words, each point (P) on the curve is P = (Px + Py), 160 + 160 = 320 bits; 32 bits are considered for the time stamp, flag, and identity. Table 3 shows the message fields and their size in bits.

**Table 3:** Message fields and their size in bits.

Description	Size
Hash function	160
Elliptic-curve cryptography	320
Random numbers	160
Timestamp	32
Identity	32
Flag	32

The exchange messages in state 1 between Idvi and IdRi are {Idvi, TVi, Psw, Pi, Ps, Pw, Pow, IdRi}, {Rr, Idvi′, HRr, HRPr*, TRi, Idvi}. state 1 requires (160 + 32 + 160 + 160 + 160 + 160 + 32) + (32 + 32 + 32 + 160 + 160 + 320 + 32 + 160 + 160 + 32 + 32) = 2016bits. In state 2, the messages exchanged between Idvi and IdRi and IdFi are {Pvar1, Pvar2, Pvar3, TVi, Idvi, Idvi′, IdRi, HRr}, {Pvar4, Pvar5, Pvar6, IdRi, TRi, Idvi}, {VAR, Vh, Ps, Pow, TVi, IdRi, Idvi, HRr}, {Idvi′, Idvi, Pow, Ps, TRi, IdRi, IdFi, vi, vp, vrf, Rv}. state 2 requires (320 + 160 + 160 + 32 + 32 + 320 + 32 + 160) + (320 + 320 + 160 + 32 + 32 + 32) + (160 + 160 + 160 + 32 + 32 + 32 + 32 + 160) + (320 + 32 + 32 + 160 + 32 + 32 + 32 + 160 + 160 + 160 + 160) = 4160bits. In state 3, {iv, pv, HFvi, Fvi, IdFi, TFi, IdRi, IdCs}, {CFi′, IdFi, IdCs, TCs}, {CFi, IdCs, TCs, IdTA} messages have been exchanged between IdFi and IdCs and IdTA. state 3 requires (32 + 32 + 160 + 32 + 32 + 32 + 32 + 32) + (160 + 32 + 32 + 32) + (32 + 32 + 32 + 32) = 768 bits. In state 4, the messages exchanged between Idvi and IdRi and IdFi are {Rf, Idvi′, Pr, TVi, IdRi}, {Idvi′*, Idvi′, Pr, TRi, IdFi}, {VIdvi′*, Idvi′, Pow, Pr, Idvisf, Idvisf′, TFi, IdRi, IdFi, FRH, BuIdVisf}, {Idvisf, Idvisf′, TFi, Idvi, Resf, BuIdVisf}. state 4 requires (160 + 320 + 32 + 32 + 32) + (320 + 320 + 32 + 32 + 32) + (32 + 320 + 32 + 32 + 32 + 160 + 32 + 32 + 32 + 160 + 32) + (32 + 160 + 32 + 32 + 160 + 32) = 2656bits. In state 5 messages {Idvi, Idvi′, TVi, OFi, Pofv1, Pofv2, Pofv3, BuIdVisf, Idvisf}, {Idvi′, Fisf, TVisf, Idvisf, IdRi},{Idvi′*, Idvisf, TRi},{Pofv4, Pofv5, Pofv6, TVisf, Idvi},{Vof, TVi, Idvisf} have been exchanged between Idvi and IdFi and Idvisf. state 5 requires (32 + 160 + 32 + 160 + 320 + 160 + 160 + 32 + 32) + (320 + 160 + 32 + 32 + 32) + (320 + 32 + 32) + (320 + 160 + 160 + 32 + 32) + (160 + 32 + 32) = 2976bits.In state 6, the messages exchanged between Idvi and IdRi and IdFi and Idvisf are {Processing result, IdIi, IdIisf}, {Completion offloading, IdIisf, IdRi, BuIdVisf},}. state 6 requires {Completion offloading, Idvi IdIisf, IdRi, BuIdVisf}, {Completion offloading, IdIisf, IdRi, IdFi, BuIdVisf}. (32 + 32 + 32) + (32 + 32 + 32 + 32) (32 + 32 + 32 + 32 + 32)(32 + 32 + 32 + 32 + 32) = 544bits.

The NSO-VFC is compared to the AKMF-IoV^38^, which consists of three states; the number of bits used in the system are 1024 bits, 1344 bits, and 1024 bits, respectively. Considering the nature of the NSO-VFC scheme and the number of phases for SO, an increase in communication costs can be expected. Table 4 shows the comparison of the communication cost and the number of bits used in each state.

**Table 4:** Comparison of Communication cost and the number of bits.

Comparison scheme	Number of messages	Total cost (in bits)
NSO-VFC		
(State 1)	2	2016
(State 2)	4	4160
(State 3)	3	768
(State 4)	5	2656
(State 5)	6	2976
(State 6)	5	544
AKMF-IoV		
(State 1)	2	1024
(State 2)	2	1344
(State 3)	2	1024

### Computation cost

To calculate the Computation cost of the NSO-VFC, we have used the expressions Tfh, Tfr, and Tfecm for the hash function with an arithmetic mean of 0.0023 and the symbol Tfecm for point multiplication of an elliptic curve with an arithmetic mean of 2.226, and the symbol Tfr for random numbers with an arithmetic mean is 0.539, the arithmetic mean of the defined symbols is calculated from reference^39^.

According to the things mentioned in “Communication cost”, the NSO-VFC consists of 6 states, considering that the 6th state does not use hash functions and random numbers, and multiplication of an elliptic curve does not have the ability to calculate in the evaluation.

The functions used in state 1 are Tfh13 + TR1 + Tfecm2 with calculation (Tfh13*0.0023 + Tfr1*0.539 + Tfecm2*2.226) the total cost is 5.020ms. The functions used in stat 2 are Tfh20 + Tfr2 + Tfecm4 with calculating (Tfh20*0.0023 + Tfr2*0.539 + Tfecm4*2.226) the total cost is 10.028 ms. In case 3, Tfh3 functions are used, and by calculating (Tfh3*0.0023), the total cost is 0.0069 ms. The functions used in state 4 are Tfh9 + Tfecm4 with calculation (Tfh9*0.0023 + Tfecm4*2.226) the total cost is 8.9247 ms. In case 3, Tfh16 + Tfr2 + Tfecm4 functions are used, and by calculating (Tfh16*0.0023 + Tfr2*0.539 + Tfecm4*2.226), the total cost is 10.0188 ms.

The results of the Computation cost of the NSO-VFC compared to AKMF-IoV scheme, which has a Computation cost of 0.0299 ms in the first case, 8.9247 ms in case 2, and 0.0299 ms in case 3, considering that AKMF-IoV has less number of cases than the NSO-VFC. It has a lower Computation cost than our NSO-VFC. The high Computation cost is justified because our NSO-VFC provides SO for the IoV environment. Table 5 shows the comparison of the Computation cost of the NSO-VFC.

**Table 5:** Comparison of computation cost.

Comparison scheme	Total cost	Total cost (ms)
NSO-VFC		
(State 1)	Tfh13 + Tfr1 + Tfecm2	5.020 ms
(State 2)	Tfh20 + Tfr2 + Tfecm4	10.028 ms
(State 3)	Tfh3	0.0069 ms
(State 4)	Tfh9 + Tfecm4	8.9247 ms
(State 5)	Tfh16 + Tfr2 + Tfecm4	10.0188 ms
(State 6)	NA	NA
AKMF-IoV		
(State 1)	Tfh13	0.0299 ms
(State 2)	Tfecm4 + Tfh9	8.9247 ms
(State 3)	Tfh13	0.299

### Security requirements comparison

Table 6 shows the comparison of the NSO-VFC in terms of security features with the AKMF-IoV. According to the table, it can be seen that both the NSO-VFC and AKMF-IoV can support the SOSF1, SOSF2, SOSF3, SOSF4, SOSF6, SOSF9, SOSF10, SOSF11, SOSF12, SOSF13, and SOSF14 security features, one of the features of NSO-VFC is that it has more steps than AKMF-IoV. However, it can provide SO and supports SOSF5, SOSF7, SOSF8, and SOSF15, which the AKMF-IoV lacks these items.

**Table 6:** Comparison of security features.

Comparison requirements	AKMF-IoV	NSO-VFC
SOSF1	Yes	Yes
SOSF2	Yes	Yes
SOSF3	Yes	Yes
SOSF4	Yes	Yes
SOSF5	No	Yes
SOSF6	Yes	Yes
SOSF7	No	Yes
SOSF8	No	Yes
SOSF9	Yes	Yes
SOSF10	Yes	Yes
SOSF11	Yes	Yes
SOSF12	Yes	Yes
SOSF13	Yes	Yes
SOSF14	Yes	Yes
SOSF15	No	Yes

SOSF1: Impersonation attack, SOSF2: MITM attack, SOSF3: Replay attack, SOSF4: Brute-force attack, SOSF5: Stolen verifier attack, SOSF6: Session key leakage attack, SOSF7: guessing attack, SOSF8: Rainbow table attack, SOSF9: mutual authentication, SOSF10: Session key agreement, SOSF11: Sniffer attack, SOSF12: Provides confidentiality, SOSF13: AVISPA, SOSF14: Informal Analysis, SOSF15: SO.

## Simulation

This section describes how the NSO-VFC is practically implemented in the Ns3 software on an Ubuntu-23.04 operating system. The simulation was conducted on a Dell 6430 laptop equipped with an Intel Core i7 processor, 8 GB RAM, and a 2 TB SSD. In the simulation scenario, 10 Road Side Units (RSUs) are utilized for 10, 20, and 30 Instances of Vehicles (IoV) with one fog and one cloud. A Random Waypoint Mobility model is employed for the movement of IoVs, with a constant speed of 60 m/s and no pauses. The RSUs, fog, and cloud nodes have a stationary mobility at 0 m/s. The simulation area measures 400*1500 units, using the Two-Ray Ground Loss model and the AODV routing protocol. The transmission power is set at 8 dBm, with the medium access control type as IEEE 802.11 and the wireless protocol as 802.11p. The communication ranges between IoV and IoV, fog node and IoV, fog node and fog, and fog to cloud are set at 50 m, 100 m, 150 m, and 300 m respectively. The simulation duration is 100 s.

### Simulation result

**Figure 12:**
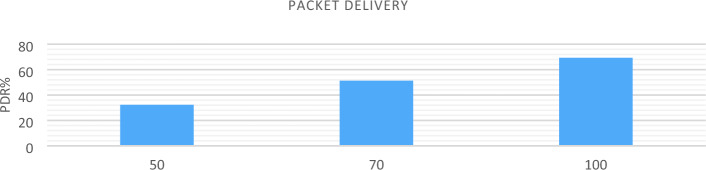
NSO-VFC packet delivery comparison.

**Figure 13:**
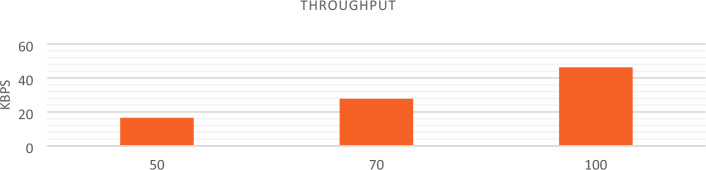
NSO-VFC throughput comparison.

**Figure 14:**
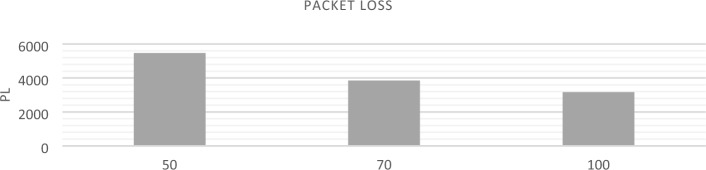
NSO-VFC packet loss comparison.

**Figure 15:**
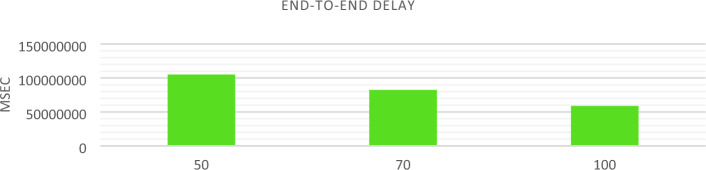
NSO-VFC end-to-end delay comparison.

AODV routing is used to implement NSO-VFC in the NS3.In AODV, a specific route is not created and maintained for every possible destination in the network, but when the source node has a packet to send, the routes are established based on the existing need. Of course, for recently used routes, routing information is stored in the routing table. Therefore, when a new route request message arrives, there is no flooding in the network because each node can only send packets to the destinations in its routing table. Considering the size of the simulation environment and taking into account that the cars are randomly arranged in the environment, there is a high probability that the cars are not next to each other or near RSU. This makes the origin unable to deliver the sent packet to its final destination and the packet gets lost on the way. For this reason, in conditions where the vehicle density is low, the end-to-end delay rate and packet loss are increasing, and the Packet delivery and Throughput are decreasing. In a situation where the traffic density is increasing, the end-to-end delay and packet loss will decrease, and the package delivery and Throughput will increase. The Figs. 12, 13, 14, and 15 compare Packet delivery, Throughput, Packet loss, and End-to-end delay, respectively. According to the simulation results, the more the number of IOVs, the higher the performance of the NSO-VFC in the network.

## Conclusion

This study investigates the issue of secure Offloading in the IoV and aims to devise a secure offloading scheme within a Fog-Cloud federation-based IoV environment. The research utilizes the Elliptic Curve Cryptography method to introduce the NSO-VFC, comprising six phases: (1) Registration phase, (2) Login and authentication phase, (3) Storage phase, (4) Request offloading phase, (5) Mutual Authentication and offloading phase, and (6) Completion of the offloading phase. Security evaluation of the NSO-VFC confirms informal and formal security. In the performance assessment of the NSO-VFC, the security aspects of the NSO-VFC outperform an AKMF-IoV scheme; however, there is an increase in communication and computational overheads due to the emphasis on security. Results obtained using NS3 indicate that with an escalation in the IoV population, the proposed scheme exhibits enhanced performance in terms of throughput and packet delivery. Future research prospects could involve the development of secure Offloading mechanisms based on blockchain networks and the Metaverse.

### Ethical approval

All procedures performed in studies without human participants. This article does not contain any studies with animals performed by any of the authors. The present study is part of a Ph.D.

## Data Availability

Data used to support this novel scheme are included within the article.
